# Quantifying the Silent Selection Pressure: Antimicrobial Stewardship and Gut Microbiome Integrity in the NICU and PICU

**DOI:** 10.3390/biomedicines14051080

**Published:** 2026-05-09

**Authors:** Fauna Herawati, Faathimah Az’zahra, Maria Anggeraini, Nur Palestin Ayumuyas, Kevin Kantono, Eko Setiawan, Rika Yulia

**Affiliations:** 1 Department of Clinical Pharmacy, Faculty of Pharmacy, University of Surabaya, Surabaya 60293, Indonesia; farzhara@gmail.com (F.A.); mariaanggeraini@gmail.com (M.A.); eko_setiawan@staff.ubaya.ac.id (E.S.); rika_y@staff.ubaya.ac.id (R.Y.); 2 Center for Medicines Information and Pharmaceutical Care (CMIPC), Faculty of Pharmacy, University of Surabaya, Surabaya 60293, Indonesia; 3 Department of Pharmacy, Rumah Sakit Umum Daerah Haji Provinsi Jawa Timur, Jalan Manyar Kertoadi, Surabaya 60116, Indonesia; yustin.ay@gmail.com; 4 School of Science, Auckland University of Technology, Private Bag 92006, Auckland 1142, New Zealand

**Keywords:** antimicrobial stewardship, anti-bacterial agents, drug utilization review, intensive care units, neonatal, intensive care units, pediatric, drug resistance, bacterial

## Abstract

**Background**: Antimicrobial stewardship in Neonatal (NICU) and Pediatric Intensive Care Units (PICUs) is complicated by rapid physiological maturation and the high vulnerability of the developing gut microbiome. Traditional metrics fails to capture the true utilization density of antibiotics in these settings. This study evaluated antimicrobial consumption patterns and alignment with the WHO AWaRe framework in two Indonesian hospitals and its impact towards patients’ length of stay. **Methods**: A retrospective multicenter study was conducted at a public hospital (Haji Hospital) and a private university hospital (HU Hospital) across 2024–2025. The study population includes all admitted patients (*n* = 315 in NICU and *n* = 12 in PICU) to calculate utilization density. Consumption was quantified using Defined Daily Dose (DDD)/100 bed-days, and qualitative assessment was performed using the WHO AWaRe classification. **Results**: Generalized linear modeling revealed that appropriate antibiotic therapy was significantly associated with a 17% reduction in hospital length of stay (β = −0.187, *p* = 0.035). At HU Hospital, PICU exhibited a seven-fold higher antimicrobial density (37.56 DDD/100) compared to NICU (5.22 DDD/100). At Haji Hospital, NICU density was 4.95 DDD/100 bed-days. Weight-normalized simulations revealed weight-based dosing disparity with low absolute DDD values in neonates mask a significant biological burden and intense selection pressure on the gut resistome due to immature renal clearance. While Haji Hospital maintained high “Access” category adherence (92.21%), HU Hospital’s PICU showed a high reliance on “Watch” agents (71.27%), specifically Ceftriaxone and Meropenem, which are known drivers of multidrug resistance. **Conclusions**: Low absolute dosing in neonates does not equate to low therapeutic density or reduced environmental pressure. The heavy use of broad-spectrum agents in the PICU acts as a primary driver for microbiome disruption. To mitigate the emergence of multidrug-resistant organisms, stewardship must transition from adult-indexed metrics (DDD) to more precise measures like Days of Therapy (DOT) and prioritize “Access” protocols to preserve microbiome integrity.

## 1. Introduction

The escalation of antimicrobial resistance (AMR) has emerged as a significant challenge to global public health security. Systematic reviews underscore a direct correlation between bacterial resistance and suboptimal prescribing, suggesting that irrational antibiotic utilization is a primary driver of resistance patterns [1]. This crisis is reflected in global mortality estimates, where millions of deaths are associated with resistant pathogens annually [2,3,4]. In Indonesia, the AMR landscape is increasingly alarming with reported resistance levels in *Escherichia coli* and *Klebsiella pneumoniae* surging between 50% and 82%, with projected mortality reaching 130,000 deaths annually if left unchecked [5]. To address these challenges, the World Health Organization Access, Watch, and Reserve (WHO AWaRe) framework serves as a strategic pillar for antibiotic stewardship to optimize the use of “Access” agents while limiting unnecessary “Watch” and “Reserve” administration [6,7].

Effective surveillance is most critical in high-vulnerability environments such as the Neonatal Intensive Care Unit (NICU) and Pediatric Intensive Care Unit (PICU) [8] where patients face a heightened risk for severe bacterial infections due to physiological fragility and underdeveloped immune systems [9]. Neonatal prescribing is uniquely complicated by dynamic pharmacokinetic (PK) and pharmacodynamic (PD) considerations [10]. Antibiotic dosing therefore must be precisely adjusted to the metabolic immaturity of the liver and kidneys, which mature significantly based on postnatal age (PNA) and gestational age [11]. For instance, as renal blood flow and the glomerular filtration rate (GFR) increase after birth, dosing intervals for standard-of-care agents like Ampicillin must be shortened transitioning from 12-h to 6-h cycles to maintain therapeutic efficacy and account for accelerated molecular clearance in older neonates [10,11].

Recent evidence also suggests that the neonatal gut acts as a critical molecular reservoir for resistance, where antibiotic use can severely disrupt the developing microbiome [12,13]. Research has found that even preterm infants who have not been directly exposed to antibiotics still carry a high number of antibiotic resistance genes (ARGs) in their gut. Such as beta-lactams and aminoglycosides due to the high-pressure NICU environment [14,15]. Postnatal antibiotic exposure further intensifies this “resistome” decreasing microbial diversity and altering the molecular mechanisms, such as horizontal gene transfer and efflux pump expression allowing bacteria to withstand antibiotic pressure [7,12]. This molecular dysbiosis underscores the urgency of stewardship to protect the integrity of the neonatal microbiome and prevent the expansion of resistant strains [11,12].

Despite the necessity of surveillance in these high-risk cohorts, data regarding the alignment of intensive care prescribing with international stewardship frameworks in Indonesian private hospitals remains limited. Therefore, this study aims to evaluate the distribution and consumption intensity of antibiotics in the NICU and PICU of a multicenter hospital network in Indonesia. By applying the WHO AWaRe classification alongside quantitative and quantitative metrics, this research seeks to identify prescribing patterns and provide a localized benchmark for antimicrobial stewardship interventions.

## 2. Materials and Methods

This study employed a multicenter, quantitative observational design with a retrospective descriptive approach to evaluate antimicrobial consumption across two distinct tertiary care settings in Surabaya, Indonesia. The investigation was conducted at Husada Utama Hospital, a private facility, focusing on the Neonatal Intensive Care Unit (NICU) and Pediatric Intensive Care Unit (PICU) and the East Java Province Haji Hospital (Haji Public Hospital) between the 2024 and 2025. By integrating secondary medical records from these diverse institutional models, the study provides a comprehensive profile of pediatric antimicrobial stewardship. The sampling strategy was designed to ensure statistical reliability and clinical relevance. The study combined multicenter samples of 321 patients (127 subjects from the private facility and 182 from the public hospital). As a retrospective study, the sample size was determined by institutional volume rather than a priori power calculation to provide a robust, representative dataset across both healthcare models. At both institutions, subjects were selected via time-limited sampling to include all patients who met the inclusion criteria of complete clinical and pharmaceutical documentation. This combined cohort facilitates a robust descriptive analysis of prescribing patterns and consumption profiles.

The data collection process involved the systematic recording of patient demographics, clinical histories, and detailed antimicrobial therapy parameters into a structured digital database. Demographic variables such as age, sex, weight, and primary diagnosis were recorded, with clinical metrics included length of stay (LOS) and admission-to-discharge timelines. For each antimicrobial intervention, specific drug agent, dosage, route of administration, and total duration of therapy were documented. All identified antibiotics were then cross-referenced with the World Health Organization (WHO) AWaRe framework to categorize them into Access, Watch, or Reserve groups, providing a qualitative lens towards prescribing appropriateness and alignment with international stewardship guidelines.

Quantitative analysis was performed using the Defined Daily Dose (DDD) per 100 bed-days and the Drug Utilization 90% (DU 90%) metrics. The DDD was utilized as a standardized technical unit of measurement to determine antimicrobial density across the populations, allowing for a rigorous comparison of consumption intensity between the NICU and PICU. Concurrently, the DU 90% method was applied to identify the specific antimicrobial agents that constituted the bulk of the clinical burden. Descriptive statistical analysis was subsequently conducted to synthesize the demographic distribution and utilization patterns, with results organized into frequency tables to highlight the primary therapeutic agents and consumption trends within these critical care environments.

Statistical analyses analyzed the data in two folds, where (1) a Pearson’s chi-square analysis was performed on the AWaRe prescribing ratios to show the significant differences between the two hospitals, and (2) a regression model was used to explore the significance of antibiotic treatment appropriateness for the patients’ length of stay. 

Additionally, for the categorical distribution of Access, Watch, and Reserve (AWaRe) status across hospitals the effect size was quantified using Cramér’s V, and standardized residuals were examined (threshold ± 1.96, *p* < 0.05) to identify specific prescribing ratios and hospitals that contributes to the overall significance.

Regression model was performed using generalized linear modeling (GLM) to explore the impact of antibiotic appropriateness which was previously determined by Appearance, Pulse, Grimace, Activity, and Respiration (APGAR) scores towards clinical outcomes, specifically the length of stay (LOS). LOS was modeled using a GLM with a Gamma distribution and log-link function which is more appropriate for positively skewed continuous healthcare data. Covariates including age, gender, weight, and height were controlled in the model. All statistical tests were two-sided, with significance defined as *p* < 0.05.

## 3. Results

Between January 2024 and October 2025, this multicenter investigation evaluated a cumulative cohort of 321 patients. The sample was distributed across the Husada Utama Hospital’s (HU Hospital) NICU (*n* = 127) and PICU (*n* = 12), alongside Haji Hospital’s neonatal units (*n* = 182).

### 3.1. Patient Cohort Profile and Clinical Baseline

Neonatal patients (0–28 days) represented the vast majority across all NICU sites. Gender distribution varied by facility: female infants were more prevalent at HU Hospital (51.18%), whereas male patients predominated in the PICU and Haji Hospital cohorts (56.59%). The leading diagnostic indications for intensive care admission were respiratory distress of the newborn (*n* = 96) and bronchopneumonia (*n* = 74). Therapeutic success was high, with a 95.05% recovery rate documented at NICU Haji Hospital, 96,06% at NICU HU Hospital, and 91,67% at PICU HU Hospital.

The clinical landscape of the NICU varies significantly across the two datasets, showing a transition from infectious to non-infectious dominance. Haji Hospital exhibited a distinct infectious profile, led by bacterial sepsis (17.02%). HU Hospital presented a more higher burden between the two categories, where pneumonia (35.43%) represents a major infectious threat, while neonatal jaundice (28.35%) remains the most prevalent non-infectious condition. This comparison highlights that, while public hospital settings may see fluctuating trends in infection control and neonatal care priorities, the private sector maintains a high prevalence of both respiratory infections and metabolic conditions like jaundice.

When comparing the overall NICU data against the PICU at HU Hospital, distinct age-related clinical priorities emerge. The NICU environments are characterized by birth-related and developmental challenges such as neonatal sepsis, respiratory distress, and jaundice. Conversely, the PICU data shifts toward neurological and acute respiratory crises, where non-infectious conditions like status epilepticus (41.67%) serve as the primary clinical driver, followed by respiratory infections (33.3%). While pneumonia remains a shared infectious burden across both units, the NICU is defined by neonatal-specific metabolic and maturity issues, whereas the PICU is dominated by critical neurological emergencies and broader pediatric respiratory infections.

To contextualize antimicrobial selection, local microbiological surveillance from Haji Hospital for 2025 was reviewed. The five most prevalent pathogens identified in blood specimens were *Staphylococcus hominis*, *Escherichia coli*, *Staphylococcus haemolyticus*, *Staphylococcus epidermidis*, and *Klebsiella pneumoniae*. Antibiotic susceptibility testing revealed that Gentamicin, Amikasin, and Meropenem maintained high sensitivity levels (≥81%) (Institutional Microbiology Report, Haji Hospital, 2025), providing a microbiological rationale for the stewardship protocols.

### 3.2. Quantitative Assessment of Antimicrobial Density

The measurement of antibiotic consumption via the DDD/100 bed-days metric revealed differences in prescribing intensity that significantly exceeded physiological predictions based on patient weight. Utilizing a weight-adjusted threshold, calculated as 5.22 DDD/100 bed-days for neonates (mean 3 kg) and 20 DDD/100 bed-days for pediatric patients (mean 12 kg), the PICU’s observed consumption of 37.56 units indicates a substantial excess, exceeding the predicted daily dose by 87.8% (17.56 units). This high-intensity profile was primarily driven by Ceftriaxone, which at 19.31 DDD/100 bed-days nearly exhausted the unit’s entire physiological threshold independently. In contrast, utilization at the HU Hospital NICU (5.22 DDD/100 bed-days) demonstrated high alignment with the predicted requirements, exhibiting a negligible variance of only 4.4%.

Intravenous Ampicillin (IV) usage illustrates the gap between real-world clinical dosing and standardized measurement metrics across different hospital settings. According to the BNF for Children (2022–2023), neonatal dosing is weight-dependent, typically requiring 30 mg/kg every 6 to 12 h. When evaluating the HU Hospital NICU, the recorded consumption was 0.99 DDD/100 bed-days, whereas the Haji Hospital NICU documented a significantly lower volume of 0.03 DDD/100 bed-days.

When these values are compared against the weight-adjusted physiological prediction for a 3 kg neonate, as calculated from the WHO adult DDD of 3 g for parenteral Ampicillin, the expected baseline is approximately 0.043 DDD/100 bed-days. The data reveals that, while the Haji Hospital NICU maintains a consumption level nearly identical to the physiological prediction (approximating 1.3× the baseline), the HU Hospital NICU exhibits a consumption intensity 23 times higher than the predicted threshold. This profound variance suggests a much higher antimicrobial pressure in the private hospital setting compared to the public one. Such findings emphasize that the DDD metric, while standardized, requires weight-based contextualization to distinguish between physiologically aligned prescribing (as seen in Haji Hospital) and high-intensity therapeutic interventions (as seen in HU Hospital).

The distribution of antimicrobial agents highlights divergent clinical strategies between hospital settings. HU Hospital NICU maintained a diversified prescribing profile across six agents (Ampicillin, Gentamicin, Ampicillin-sulbactam, Amikacin, Meropenem, and Cefixime) within the Drug Utilization 90% (DU 90%) segment. Conversely, Haji Hospital documented a substantial surge in Gentamicin alone, reaching 1.59 DDD/100 bed-days and accounting for 32.12% of the unit’s total volume. These findings suggest that, while HU Hospital NICU adheres closely to weight-adjusted dosing, Haji Hospital NICU and HU Hospital PICU exhibit significant antimicrobial pressure. This is particularly evident in PICU’s disproportionately narrow and intense focus on third-generation cephalosporins, which far surpasses the standard daily maintenance dose expected for the pediatric population.

### 3.3. AWaRe Categorization and Prescribing Ratios

According to WHO AWaRe’s (2025) classification revealed a pronounced contrast in stewardship performance, with the public facility (Haji Hospital) demonstrating a significantly higher alignment with global benchmarks by achieving an “Access” group proportion of 92.21% and a robust Access-to-Watch ratio of 11.84. In contrast, the private university hospital (HU Hospital) encountered a “Watch” group challenge. HU Hospital NICU remained below the 60% Access target at 51.71%, while the PICU exhibited a heavy dependence on restricted agents, with the “Watch” category comprising 71.27% of all prescriptions. This disparity was further reflected in the PICU’s low Access-to-Watch ratio of 0.28, emphasizing the need for targeted stewardship interventions to reduce reliance on broad-spectrum antimicrobials in pediatric critical care.

Analysis of the AWaRe distribution across hospital settings revealed a significant association between institutional type and antibiotic category (χ^2^(6) = 52.93, *p* < 0.001). The strength of this association was moderate to strong (Cramér’s V = 0.286), indicating distinct prescribing behaviors between the public and private sectors.

### 3.4. Statistical Correlation of Appropriateness and Clinical Outcomes

In the HU Hospital cohort (NICU and PICU), LOS regression model demonstrated modest but acceptable explanatory power (Pseudo R^2^ = 0.266). Notably, with appropriate antibiotics, β = −0.187 (95% CI: −0.361 to −0.013, *p* = 0.035), corresponding to a LOS ratio of 0.83 (95% CI: 0.70 to 0.99). This translates to approximately 17% reduction in LOS with appropriate antibiotic use. Patient weight showed a significant negative relationship with LOS (β = −0.442; 95% CI: −0.587 to −0.297, *p* < 0.001)), corresponding to a LOS ratio of 0.64 (95% CI: 0.56 to 0.74). To note that the appropriateness of antibiotic selection based on APGAR score was 62% in HU Hospital and 74% in Haji Hospital.

## 4. Discussion

### 4.1. Demographic Profile and Clinical Baseline

The predominant proportion of neonates (96.26%) in this multicenter cohort highlights the acute physiological vulnerability of this population, particularly for peripartum infections and complications of prematurity [16,17]. These findings align with neonatal admission trends in other developing regions where early-life susceptibility drives intensive care utilization [16,18]. This demographic is also defined by developmental pharmacokinetic ontogeny, where the rapid maturation of renal function and hepatic enzymes creates a highly dynamic environment for drug clearance [10,11]. Specifically, the molecular transition from fetal CYP3A7 to adult-like CYP3A4 activity significantly alters the metabolic pathways of various xenobiotics during the first weeks of life. Accordingly, the high prevalence of respiratory distress syndrome and bronchopneumonia (*n* = 170) requires empirical interventions that must be precisely adjusted to these evolving metabolic rates in order to avoid systemic toxicity while ensuring therapeutic efficacy [19,20]. Furthermore, the management of respiratory conditions in this period occurs during a critical developmental window for microbiome establishment especially as the neonatal gut is a fundamental reservoir for the metagenomic resistome [12]. Early-life exposure to broad-spectrum “Watch” group antibiotics can lead to a profound depletion of microbial alpha diversity and the molecular expansion of antibiotic resistance genes (ARGs) [12]. In this study, while the high recovery rate (over 90%) suggests effective acute management, the extensive dependence on clinical diagnosis for pneumonia reinforces the need for stewardship strategies that prioritize “Access” agents. Such a shift is essential not only to combat systemic resistance but also to protect the fragile microbial ecosystem of the neonatal gut from the long-term molecular selection pressure and horizontal gene transfer associated with broad-spectrum cephalosporins [12,19].

### 4.2. Quantitative Consumption and Therapeutic Density (DDD)

The seven-fold disparity in antimicrobial density between the PICU (37.56 DDD/100 bed-days) and the NICU (5.22 DDD/100 bed-days) is largely a result of weight-based dosing disparity. WHO’s DDD is standardized for a 70 kg adult, which fails to accurately capture the pharmacological requirements of a 3 kg neonateand underestimates their actual drug exposure [21]. Consequently, as pediatric patients grow and their weight increases toward adult levels, the necessary rise in absolute doses mathematically inflates the DDD values making usage in the PICU appear significantly higher.

Weight-normalized simulations demonstrate that the modest DDD values in the NICU masks a substantial antimicrobial burden. This is clearly evidenced by the age-dependent escalation of Ampicillin regimens; while a child may receive a standard 250 mg dose four times daily, a neonate’s regimen is strictly governed by postnatal age to account for evolving clearance rates. In the first week of life, Ampicillin is administered at 30 mg/kg every 12 h, with the frequency increasing to every 8 or 6 h as the infant approaches 28 days of life. Although a 3 kg neonate may register a DDD nearly 7.5 times lower than a 15 kg toddler, the relative biological pressure per kilogram remains elevated due to reduced renal clearance capacity. Given that neonatal GFR is much lower than pediatric reference values, even relatively small doses can impose considerable metabolic stress. This systemic exposure indicates that the neonatal “resistome” faces aggressive selection pressure, even when quantitative metrics appear low. Specifically, the transition from fetal CYP3A7 to adult-like CYP3A4 activity, alongside accelerated GFR, necessitates precise titration in the first week of life [10,11]. In contrast, the PICU’s utilization of Ceftriaxone (19.31 DDD/100 bed-days) signals a migration toward broad-spectrum “Watch” agents, which facilitate metagenomic disruption and the expansion of multidrug-resistant organisms (MDROs) [12,22,23].

Within NICU, the prioritized use of Gentamicin (1.59 DDD/100 bed-days) reflects its role as a standard-of-care empirical agent for sepsis [24]. However, data reveals an acute dependence on Aminoglycosides that introduces severe molecular liabilities. At Haji hospital, the 13 mg daily dose for a 2.6 kg neonate exceeds the weight-adjusted equivalent of an adult DDD (9 mg/day), confirming its high therapeutic density. Beyond its efficacy, high-dose Gentamicin acts as a primary trigger for iatrogenic injury [25]. It is important to note that intracellular accumulation of aminoglycosides triggers irreversible hair cell apoptosis, leading to permanent sensorineural hearing loss, a process validated in pediatric audiological surveillance [26,27]. Intracellular accumulation then activates parallel pathways of necroptosis and apoptosis, fueled by reactive oxygen species and caspase signaling [26,28]. Because these mechanosensory cells lack regenerative capacity, the resulting sensorineural hearing loss is permanent [27]. Additionally, presence of lipopolysaccharide (LPS) during sepsis can exacerbate this ototoxicity, further compromising neonatal hearing outcomes [29]. These cytotoxic risks demonstrates the need of stewardship frameworks that favor weight-normalized safety profiles over traditional, adult-indexed consumption data.

### 4.3. AWaRe Framework Alignment and Global Stewardship

A qualitative audit using the WHO AWaRe classification revealed a disparity in stewardship performance between the two hospitals, characterized by the public hospital achieving a 92.21% “Access” group proportion and a robust Access-to-Watch ratio of 11.84 in 2025. In contrast, the private hospital struggled to meet global benchmarks, with the NICU falling below the 60% “Access” target (51.71%) and the PICU exhibiting a concerning 71.27% of “Watch” category agents. This intensive broad-spectrum reliance is mirrored in pediatric critical care units similarly in Brazil and South Africa, as well as recent evidence from Southeast Asia indicating high rates of “Watch” and “Reserve” antibiotic utilization among neonates in the region [30,31,32,33]. Beyond administrative metrics, gut resistome theory suggests that this prolonged dependence on cephalosporins and carbapenems serves as a selective driver that disrupts early microbiome assembly, depleting microbial alpha diversity and expanding the intestinal reservoir of antibiotic resistance genes (ARGs) [14]. Accordingly, the high Access-to-Watch ratio observed at the public facility represents a critical ecological intervention. By prioritizing “Access” agents, the facility not only aligns with global stewardship goals but actively preserves the integrity of the developing neonatal microbiome against the long-term selection pressure and horizontal gene transfer of multidrug-resistant organisms.

Our inclusion of the 2025 antibiogram data (Table S6) confirms that, while non-susceptibility to first line “Access” agents like Ampicillin was observed in *E. coli*, Gram-positive pathogens maintained high sensitivity (≥81%) to alternative “Access” agents such as Gentamicin and Amikacin. This local sensitivity pattern justifies the “Access-prioritized” stewardship strategies utilized at Haji Hospital and emphasizes that clinical guidelines must be calibrated against localized microbiological surveillance.

Furthermore, our statistical findings indicate that antibiotic appropriateness is a key modifiable determinant of hospital length of stay (LOS). The observed significant ~17% reduction in LOS associated with appropriate therapy suggests that adherence to stewardship protocols not only improves individual patient outcomes but also significantly reduces the institutional resource burden. This underscores that effective stewardship in Indonesian intensive care settings provides a dual benefit: enhancing clinical safety while achieving economic efficiency by minimizing prolonged hospitalizations.

### 4.4. Limitations

This investigation is subject to several limitations that warrant consideration. Its retrospective design limits the ability to perform a detailed assessment of additional clinical information, such as correlations between specific antimicrobial selections, real-time laboratory culture results, and adherence to bedside clinical pathways. In addition, Defined Daily Dose (DDD) provides a standardized metric for cross-ward comparison, its derivation from adult-centric dosing (based on a 70 kg standard) introduces a mathematical oversimplification bias when applied to neonatal and pediatric cohorts as DDD may not fully reflect the biological burden or the precision of weight-tailored regimens necessitated by dynamic PK ontogeny and the evolving renal clearance of early life [28]. Additionally, the lack of prospective microbiological data limits our ability to directly link prescribing patterns to changes in the gut microbiome. Future research should focus on measuring Days of Therapy (DOT), which is independent of adult dosing standards, and incorporating longitudinal metagenomic analysis of fecal samples would provide a more robust understanding of how antimicrobial stewardship can actively mitigate the expansion of the neonatal resistome within the intensive care landscape [30].

## 5. Conclusions

This multicenter analysis demonstrates that the seven-fold difference in antimicrobial density between the PICU and NICU is driven by weight-based dosing requirements. Although absolute doses appear lower in neonates, weight-normalized simulations reveal a substantial biological burden as drug exposure is concentrated within a smaller mass and governed by immature renal clearance. Neonatal resistome remains under aggressive selection pressure despite adult-indexed metrics, suggesting lower consumption, while the high utilization of “Watch” agents in the private sector (71.27%) acts as a selective driver for microbiome disruption and the expansion of resistance genes. The significant relationship between appropriate therapy and reduced length of stay (β = −0.187) proving that stewardship is one of the primary drivers of clinical recovery speed. It is recommended that healthcare facilities must institutionalize “Access-prioritized” protocols and transition toward weight-normalized monitoring, ensuring that stewardship evolves to treat the preservation of the developing metagenome and the prevention of iatrogenic molecular injury as primary clinical outcomes that account for the rapid physiological maturation of intensive care patients.

## Data Availability

The original contributions presented in this study are included in the article/Supplementary Material. Further inquiries can be directed to the corresponding author.

## Supplementary Materials

The following supporting information can be downloaded at: https://www.mdpi.com/article/10.3390/biomedicines14051080/s1, Table S1: Patient Demographic Characteristics; Table S2: Diagnosis or condition of NICU and PICU; Table S3: DDD/100 Bed-days PICU; Table S4: DDD/100 Bed-days NICU; Table S5: AWaRe Categorization Between Hospitals; Table S6: Antibiotic Sensitivity Profiles (Antibiogram) of Blood Specimens in Haji Hospital, 2025.

## Abbreviations

The following abbreviations are used in this manuscript:

AMR	Antimicrobial Resistance
ARGs	Antimicrobial Resistance Genes
AWaRe	Access, Watch, Reserve
DDD	Defined Daily Dose
DOT	Days of Therapy
DU	Drug Utilization
GFR	Glomerular Filtration Rate
LOS	Length of Stay
LPS	Lipopolysaccharide
MDROs	Multidrug-Resistant Organisms
NICU	Neonatal Intensive Care Unit
PICU	Pediatric Intensive Care Unit
PK	Pharmacokinetics
PNA	Postnatal Age
WHO	World Health Organization
