# Supplementary material for: Quantifying the Silent Selection Pressure: Antimicrobial Stewardship and Gut Microbiome Integrity in the NICU and PICU
Source: Biomedicines. 2026 May 9;14(5):1080. doi: 10.3390/biomedicines14051080 (PMC13203881; doi:10.3390/biomedicines14051080)
Supplement: Supplementary file 1 [file biomedicines-14-01080-s001.zip › biomedicines-4253336-supplementary.pdf]

biomedicines

Table S1. Patient Demographic Characteristics

Variable	NICU (N=315)		PICU (N=12)
	**HU Hospital (N=127)**	**Haji Hospital (N=182)**	**HU Hospital (N=12)**
**Gender:**			
Female	65 (51.18)	79 (43.41)	5 (41.67)
Male	62 (48.82)	103 (56.59)	7 (58.33)
**Age:**			
0 - 28 days	127 (100.00)	182 (100.00)	-
Mean ± SD	0.00 ± 0.00	6.40 ± 7.23	-
Median (min-max)	0.00 (0-28)	4.00 (0-28)	-
29 days - <12 months	-	-	4 (33.33)
Mean ± SD	-	-	5.25 ± 3.42
Median (min-max)	-	-	4.5 (2-10)
12 - 59 months	-	-	3 (25.00)
Mean ± SD	-	-	3.27 ± 0.40
Median (min-max)	-	-	3.5 (2.7-3.6)
5 - 18 years	-	-	5 (41.67)
Mean ± SD	-	-	8.56 ± 2.30
Median (min-max)	-	-	8.9 (5.1-11.6)
Total (age)	127	182	12
Mean ± SD	0.00 ± 0.00 (day)	0.00 ± 0.00 (day)	53.23 ± 47.17 (months)
Median (min-max)	0.00 (0-28)	0.00 (0-28)	42.6 (2-139.2)
**Weight (kg)**			
Mean ± SD	3.00 ± 0.66	2.58 ± 0.79	13.54 ± 8.05
Median (min-max)	3.07 (0.58-4.19)	2.50 (0.75-4.10)	11.3 (3.8-33)
Not available	34	2	1
**Length of stay (LOS)**	808	1933	65
Mean ± SD			
Median (min-max)	6.33 ± 4.98	10.32 ± 6.81	5.42 ± 3.84
	4 (1-32)	9 (1-44)	4 (1-16)
Outcome			
Recovery	122 (96.06)	173 (95.05)	11 (91.67)
Died	5 (3.94)	5 (2.75)	-
Referred	-	4 (2,20)	1 (8.33)

Table S2. Diagnosis or condition of NICU and PICU

Setting	Top Infectious Drivers	Top Non-Infectious Drivers
HU Hospital NICU	Pneumonia (35.43%)	Neonatal Jaundice (28.35%)
Haji Hospital NICU	Bacterial Sepsis (17.02%)	Respiratory Distress (20.74%)
HU Hospital PICU	Respiratory Infections (33.3%)	Status Epilepticus (41.67%)

Table S3. DDD/100 Bed-days PICU

					HU Hospital		
**ATC code**	**Antibiotic Name**	**Route**	**DDD WHO (g)**	**BNF-C 2022–2023 Dosing Guidelines**	**Total Dose (g)**	**Total DDD**	**DDD/100 Bed-days**
J01DD04	Ceftriaxone	IV	2	50–80mg/kg once daily	25.1	12.55	19.31
J01GB03	Gentamicin	IV	0.24	2.5mg/kg every 8 hours	1.06	4.42	6.79
J01DH02	Meropenem	IV	3	10–20mg/kg every 8 hours	10.95	3.65	5.62
J01AA12	Tigecycline	IV	0.1	Child <8 years: Not available	0.16	1.6	2.46
J01CA01	Ampicillin	IV	6	250mg4 times a day; increased if necessary up to 30mg/kg 4 times a day	6	1	1.54
J01DD08	Cefixime	PO	0.4	5-9 years: 200 mg daily	0.38	0.95	1.46
J01DD62	Cefoperazone -sulbactam	IV	4	Not available	1	0.25	0.38
			Total			24.42	37.56

* Total Length of stay 65 days

https://doi.org/10.3390/xxxxx

biomedicines

MDPI

Table S4. DDD/100 Bed-days NICU

ATC code	Antibiotic Name	Route	DDD WHO (g)	BNF-C 2022–2023 Dosing Guidelines	HU Hospital Total DDD	HU Hospital DDD/100 Bed-days	Haji Hospital Total DDD	Haji Hospital DDD/100 Bed-days
J01CA01	Ampicillin	IV	6	30mg/kg every 12 hours (neonate up to 7 days); every 8 hours (neonate 7 to 20 days); every 6 hours (neonate 21 to 28 days)	8.05	0.99	0.63	0.03
J01GB03	Gentamicin	IV	0.24	5mg/kg every 36 hours (neonate up to 7 days); every 24 hours (neonate 7 to 28 days)	7.42	0.92	30,78	1.59
J01CR01	Ampicillin-sulbactam	IV	6	30mg/kg every 12 hours (neonate up to 7 days); every 8 hours (neonate 7 to 20 days); every 6 hours (neonate 21 to 28 days)	6.22	0.77	27.97	1.45
J01GB06	Amikacin	IV	1	15mg/kg every 24 hours; loading dose: 10 mg/kg then 7,5 mg/kg every12 hours	5.51	0.68	18.78	0.97
J01DH02	Meropenem	IV	3	20 mg/kg every 12 hours (neonate up to 7 days); 20 mg/kg (neonate 7 to 28 days)	5.13	0.63	15.87	0.82
J01DD08	Cefixime	PO	0.4	Child 6-11 months:75 mg daily; 1-4 years: 100 mg daily	4.08	0.50	-	-
J01DD62	Cefoperazone-sulbactam	IV	4	Not available	2.46	0.30	0.73	0.04
J01MA12	Levofloxacin	IV	0.5	Apply every 2 hours for first 2 days, to be applied maximum 8 times a day, then apply 4 time a day for 3 days.	1.04	0.13	-	-
J01XA01	Vancomycin	IV	2	15 mg/kg every 24 hours	0.98	0.12	-	-
J01DD02	Ceftazidime	IV	4	25 mg/kg every 24 hours (neonate up to 7 days); every 12 hours (neonate 7 to 20 days); every 8 hours (neonate 21 to 28 days)	0.53	0.07	-	-
J01DD01	Cefotaxime	IV	4	20-50 mg/kg once daily (neonate up to 15 days); 50-80 mg/kg once daily (neonate 15 to 28 days)	0.37	0.05	0.605	0.03
J01DD04	Ceftriaxone	IV	2	20-50 mg/kg once daily (neonate up to 15 days); 50-80 mg/kg once daily (neonate 15 to 28 days)	0.20	0.03	-	-
J01XD01	Metronidazole	IV	1.5	loading dose 15 mg/kg, followed by 7.5mg/kg after 24 hours, then 7.5mg/kg daily usually treated for a total duration of 7 days	0.17	0.02	0.35	0.02
J01FA10	Azithromycin	PO	0.3	12 mg/kg once daily	0.10	0.01	-	-
					42.26	5.22	95.715	4.95

* Total Length of stay HU Hospital 808 days; Haji Hospital 1933 days. WHO AWaRe classification 2025, BNF-C 2022-2023

biomedicines

MDPI

Table S5. AWaRe Categorization Between Hospitals

Category	WHO Target	NICU (HU Hospital)	NICU (Haji Hospital)	PICU (HU Hospital)
Access	≥60%	51.71%<br>Ampicillin<br>Gentamicin	92.21% (+)<br>Ampicillin-Sulbactam<br>Gentamicin	22.18%<br>Ampicillin<br>Gentamicin
Watch	Monitor	46%<br>Amikacin<br>Meropenem	7.79% (-)<br>Meropenem<br>Cefotaxime	71.27%<br>Ceftriaxone<br>Meropenem
Reserve	Minimize	2.29%<br>Vancomycin	-	6.55%<br>Tigecycline

* * + & - indicates significantly higher (positive residuals) and significantly lower (negative residuals) respectively at 5% level.

Table S6. Antibiotic Sensitivity Profiles (Antibiogram) of Blood Specimens in Haji Hospital, 2025.

Pathogen Isolate	N	GEN (%)	AMK (%)	MEM (%)	VAN (%)	LNZ (%)
Staphylococcus hominis	125	99%	-	72%	99%	100%
Escherichia coli	45	68%	100%	100%	-	100%
Staphylococcus haemolyticus	34	43%	-	43%	100%	100%
Staphylococcus epidermidis	26	64%	-	64%	100%	100%
Klebsiella pneumoniae	19	58%	100%	100%	-	100%

* Legend for Table. 6: ≥ 81% High Sensitivity, 61% - 80% Moderate Sensitivity, 0% - 60% Low Sensitivity/Resistance, N: Number of total isolates tested.

Standard Code - Full English Antibiotic Name:
AMK Amikacin
GEN Gentamicin
LNZ Linezolid
MEM Meropenem
VAN Vancomycin
